# De novo mutations of *KIAA2022* in females cause intellectual disability and intractable epilepsy

**DOI:** 10.1136/jmedgenet-2016-103909

**Published:** 2016-06-29

**Authors:** Iris M de Lange, Katherine L Helbig, Sarah Weckhuysen, Rikke S Møller, Milen Velinov, Natalia Dolzhanskaya, Eric Marsh, Ingo Helbig, Orrin Devinsky, Sha Tang, Heather C Mefford, Candace T Myers, Wim van Paesschen, Pasquale Striano, Koen van Gassen, Marjan van Kempen, Carolien G F de Kovel, Juliette Piard, Berge A Minassian, Marjan M Nezarati, André Pessoa, Aurelia Jacquette, Bridget Maher, Simona Balestrini, Sanjay Sisodiya, Marie Therese Abi Warde, Anne De St Martin, Jamel Chelly, EuroEPINOMICS-RES MAE working group, Ruben van ‘t Slot, Lionel Van Maldergem, Eva H Brilstra, Bobby P C Koeleman

**Affiliations:** 1 Department of Medical Genetics, University Medical Center Utrecht, Utrecht, The Netherlands; 2 Division of Clinical Genomics, Ambry Genetics, Aliso Viejo, California, USA; 3 Epilepsy Unit, Inserm U 1127, CNRS UMR 7225, Sorbonne Universités, UPMC Univ Paris 06 UMR S 1127, Institut du Cerveau et de la Moelle épinière, ICM, AP-HP, Hôpital de la Pitié Salpêtrière, Centre de reference épilepsies rares, Paris, France; 4 Neurogenetics Group, Department of Molecular Genetics, VIB, Antwerp, Belgium; 5 Laboratory of Neurogenetics, Institute Born-Bunge, University of Antwerp, Antwerp, Belgium; 6 Danish Epilepsy Centre, Dianalund, Denmark; 7 Institute for Regional Health Services, University of Southern Denmark, Odense, Denmark; 8 New York State Institute for Basic Research in Developmental Disabilities, Staten Island, New York, USA; 9 Albert Einstein College of Medicine, Bronx, New York, USA; 10 Division of Neurology, The Children's Hospital of Philadelphia, Philadelphia, Pennsylvania, USA; 11 NYU Comprehensive Epilepsy Center, New York University Langone Medical Center, New York, New York, USA; 12 Department of Pediatrics, Division of Genetic Medicine, University of Washington, Seattle, Washington, USA; 13 Department of Neurology, UZ Leuven, Leuven, Belgium; 14 Department of Neurosciences, Rehabilitation, Ophthalmology, Genetics, Maternal and Child Health, G. Gaslini Institute, University of Genoa, Genova, Italy; 15 Centre de génétique humaine, Université de Franche-Comté, Besançon, France; 16 Division of Neurology, Department of Paediatrics, The Hospital for Sick Children and University of Toronto, Toronto, Canada; 17 Genetics Program, North York General Hospital and Prenatal Diagnosis & Medical Genetics, Mt. Sinai Hospital, Toronto, Canada; 18 University of Fortaleza, Fortaleza, Brazil; 19 Service de génétique, GHU Pitié-Salpêtrière, Université Pierre et Marie Curie, Paris, France; 20 UCL Institute of Neurology, London, UK; 21 Epilepsy Society, Bucks, UK; 22 Service de Pédiatrie, Hôpitaux Universitaires de Strasbourg, Strasbourg, France; 23 Institut de Génétique et de Biologie Moléculaire et Cellulaire, Université de Strasbourg, Illkirch, France; 24 Service de Diagnostic Génétique, Hôpital Civil de Strasbourg, Hôpitaux Universitaires de Strasbourg, Strasbourg, France

**Keywords:** Clinical genetics, Epilepsy and seizures, <i>KIAA2022</i>, X-linked

## Abstract

**Background:**

Mutations in the *KIAA2022* gene have been reported in male patients with X-linked intellectual disability, and related female carriers were unaffected. Here, we report 14 female patients who carry a heterozygous de novo *KIAA2022* mutation and share a phenotype characterised by intellectual disability and epilepsy.

**Methods:**

Reported females were selected for genetic testing because of substantial developmental problems and/or epilepsy. X-inactivation and expression studies were performed when possible.

**Results:**

All mutations were predicted to result in a frameshift or premature stop. 12 out of 14 patients had intractable epilepsy with myoclonic and/or absence seizures, and generalised in 11. Thirteen patients had mild to severe intellectual disability. This female phenotype partially overlaps with the reported male phenotype which consists of more severe intellectual disability, microcephaly, growth retardation, facial dysmorphisms and, less frequently, epilepsy. One female patient showed completely skewed X-inactivation, complete absence of RNA expression in blood and a phenotype similar to male patients. In the six other tested patients, X-inactivation was random, confirmed by a non-significant twofold to threefold decrease of RNA expression in blood, consistent with the expected mosaicism between cells expressing mutant or normal *KIAA2022* alleles.

**Conclusions:**

Heterozygous loss of *KIAA2022* expression is a cause of intellectual disability in females. Compared with its hemizygous male counterpart, the heterozygous female disease has less severe intellectual disability, but is more often associated with a severe and intractable myoclonic epilepsy.

## Introduction

*KIAA2022* is a known X-linked intellectual disability (XLID) gene, with pathogenic variants causing severe intellectual disability (ID) in males. Other, more variable features include epilepsy, postnatal growth retardation, autistic behaviour, strabismus and dysmorphic facial features. The first description of alterations in this gene causing ID was in two related male patients, where both *KIAA2022* and *P2YR8* were interrupted by a pericentric inversion of the X chromosome (Inv X(p22;p13.2)), as reported by Cantagrel *et al*.^1^ One breakpoint was mapped to the first intron of *KIAA2022* and was predicted to disrupt the gene. A complete loss of *KIAA2022* expression in lymphocytes was shown. Other reported *KIAA2022* pathogenic variants include a microduplication of exon 1, a duplication of the entire gene and several truncating mutations, all leading to reduced *KIAA2022* expression.^2–5^ Limited data about *KIAA2022* gene function are available, but it is thought to have an important role in early brain development.^1^ ^2^ ^6–8^

Female relatives carrying the *KIAA2022* disruptions identified in reported males were all unaffected.^1^ ^2^ ^5^ Nevertheless, a few affected female patients have recently been described.^9–11^ Here, we report 14 female patients with heterozygous de novo mutations of *KIAA2022*. All mutations result in a frameshift or premature stop codon, predicting complete loss of function (LOF) of the protein. Twelve patients had epilepsy, of which eleven generalised, and all but one patient had mild to severe ID. These data strongly suggest that pathogenic *KIAA2022* variants can lead to a phenotype in males and in females.

## Methods

### Patients

All 14 reported females were selected for genetic testing because of their substantial developmental problems and/or epilepsy. In 10 patients, exome sequencing was performed, using methods that were described previously,^12–14^ of which 9 were diagnostic and 1 was in a research setting (patient 4).^i^ In patient 9, a whole genome sequencing was performed in a research setting, as previously described.^15^ Patient 5 was part of a research series of 209 cases with Dravet(-like) syndrome or myoclonic atonic epilepsy who were selected for candidate gene screening with a gene panel using molecular inversion probes (MIPS) as described previously.^16^ Patient 8 was diagnosed after a diagnostic array CGH (Agilent 180 k chip) indicated a microdeletion disrupting *KIAA2022*. Patient 11 was diagnosed after a *KIAA2022* mutation was identified with her two sons with ID, through sequencing of a diagnostic ID-related gene panel. X-inactivation and expression studies were performed when possible (X-chromosome inactivation, XCI: patients 1, 2, 3, 4, 6, 8, 9; expression studies: patients 1, 3, 4, 5, 6).

See online supplemental material for extensive details on the molecular analyses (see online supplementary appendix 1).

10.1136/jmedgenet-2016-103909.supp1 Supplementary material

Detailed clinical data were collected from medical records. The study was approved by the ethical committees of the respective local institutions.

### Literature search

A literature study was carried out in the PubMed database to identify previously described patients with *KIAA2022* mutations. Resulting articles and their references were screened for reported patients with *KIAA2022* mutations.

## Results

### Clinical description

Clinical characteristics of the 14 new and 3 previously described female patients with de novo *KIAA2022* mutations are summarised and compared with the clinical characteristics of previously reported males in tables 1 and 2. Table 3 gives further details with respect to the epilepsy phenotype of the female patients. See supplemental material for extensive clinical descriptions of each patient (see online supplementary appendix 2). In summary, 12 out of the 14 new patients had intractable epilepsy with myoclonic and/or absence seizures. In 11 patients, epilepsy was generalised. Thirteen patients had mild to severe ID. Developmental delay preceded epilepsy onset in six individuals. Behavioural problems, such as autism, aggression and hyperactivity, were present in 10 patients. Other findings were hypotonia, neonatal feeding difficulties, microcephaly and mild dysmorphic facial features.

**Table 1:** Clinical description of female cases presented carrying *KIAA2022* mutations

Patient	1	2	3	4	5	6	7	8	9	10	11	12	13	14	Ref. 9	Ref. 10	Ref. 11
Age (years)	26	9	25	11	36	2,5	9	18	5,5	2,3	53	11	51 (deceased)	8,5	26	13	17
Mutation	c.4185del p.Lys 1396fs	c.438 C>A p.Cys 146*	c.2042del p.Gly 681fs	c.964C>T p.Arg322*	c.2201_2202 delAA p.Lys734 Serfs*24	c.1441 C>T p.Arg481*	c.3053-3066del14 p.Gly1018 Aspfs*2	del exon1	c.1582delA p.Arg 528Glufs*4	c.1882 C>T p.Arg 628*	c.2725del p.Ala909Profs*13	c.652C>T p.Arg218*	c.952C>T p.Gln318*	c.3596_3597 insA p.Lys 1199Asnfs	46,X,t(X;3) (q13;q11)	c.1882C>T p.Arg628*	c.964C>T p.R322*
XCI	57:43 (random)	70:30 (random)	52:48 (random)	63:37 (random)		100% skewing		62:38 (random)	64:36 (random)						100% skewing	65:35 (random)	73:27 (borderline skewed)
*KIAA2022* expression (ratio)	0.000624456		0.00029598	0.000355424	0.000256076	Absent									Absent		
Walking age (months)	24	18	19	15	14,5	−	12	24	14	19	12	15	24	18	?	?	12–18
Language skills	Sentences	Sentences	Sentences	Sentences	Sentences	Absent	Full sentences	Absent	150 words	5 words	Normal	Two-word phrases	Single simple words, no sentences	Simple sentences	Absent	?	Two words
ID	+	+	+	+	+	+	+	+	+	+	−	+	+	+	+	+	+
Degree of ID	+	+	+/−	+	+ to ++	+	+/− to +	+ to ++	+	+/−	−	+	++	+	++	+/−	++
Age at first notice of delay (months)	18	8	From birth	30	12–24	3	35	15	12–18	7	−	<20	12–24	12–18	?	?	18
Autistic behaviour	+	+	−	+	−	−	+	+	−	−	−	+	−	−	+	?	+
Other neurobehavioural problems	−	Aggression hyperactive	Tantrums hyperactive	ADHD	Attention-deficit hyperactive	−	+	Tantrums hyper-active	Hyperactive, impulse control difficulties	−	−	Severe ADHD, impulse control disorder	−	Opposition	−	?	Repetitive behaviours, aggression, hyperactive
Seizures	+	+	+	+	+	−	+	+	+	−	+	+	+	+	−	−	+
Neurological examination	Normal	Normal	Normal	Normal	Normal	Hypotonia	Normal	Normal	Normal tone ataxic gait	Normal	Normal	Hypotonic, broad-based gate	Normal	Normal	?	?	Normal
Growth retardation, prenatal	−	−	−	<−2SD	−	−2 SD	−	−	−	−	−	−	−	−	p3–p10	?	−
Growth retardation, postnatal	−	−	−	−	−	+	−	−	−	−	−	−	+	−	+	−	+
Obesity	+	+	+	−	−	−	−	+	−	−	−	−	+	−	−	?	−
Microcephaly	−	−	−	−	−	+	−	−	−	−	−	−	−	−	+	?	+
Dysmorphisms	−	+	−	−	−	+	−	−	−	−	−	−	+	−	+	−	+
Joint laxity	+	−	−	−	−	−	−	−	−	+	−	−	−	+	−	?	
Hypotonia	−	−	−	−	−	+	Very mild	−	−	+	−	+	−	Mild	−	?	−
Additional medical problems	Hip dysplasia	GER	Neonatal feeding difficulties	−	−	GER	−	−	Otitis media, PFO	−	IDDM, horse-shoe kidney	Cardiac rhabdo-myoma, TSC 1 and 2 negative	−	Pulmonary stenosis	Primary amenorrhoea, hyperglycaemia	?	−
MRI brain	Normal	Normal	Normal	Normal	Normal	Normal	Normal	Normal	Normal	Normal	?	Normal	Normal	Frontal atrophy (3 years)	Morphological alterations of temporal lobes	?	Status after corpus callosotomy surgery

?, unknown.

ADHD, attention deficit hyperactivity disorder; GER, gastroeosophageal reflux; ID, intellectual disability; TSC, tuberous sclerosis complex; XCI, X-chromosome inactivation.

**Table 2:** Clinical description of previously reported males carrying *KIAA2022* mutations

Family	Family 1^1^ ^2^		Family 2^2^		Family 3^2^	Family 4^2^			Family 5^3^	Family 6^3^
Patient	1	2	3	4	5	6	7	8	9	10
Age (years)	13	20	6	4	8	14	10	40	3	5
Mutation	InvX	InvX	Ser1200fs	Ser1200fs	Exon 1 dup	Arg62fs	Arg62fs	Arg62fs	Gln705*	Arg322*
XCI	n.a.	n.a.	n.a.	n.a.	n.a.	n.a.	n.a.	n.a.	n.a.	n.a.
*KIAA2022* expression	Absent	Absent	?	?	40%	?	?	?	?	?
Walking age (months)	36	36	34	48	17	18	18,5	14	−	48
Language skills	Absent	Absent	Rudimentary	Absent	Rudimentary	Delayed	Poor	Poor	Absent	Absent
ID	+	+	+	+	+	+	+	+	+	+
Degree of ID	++	++	++	++	+/−	+	++	+	++	++
Age first notice of delay (months)	0–12	0–12	0–12	0–12	?	?	?	36	0–12	3
Autistic behaviour	+	+	+	+	+	−	+	−	−	+
Other neurobehavioural problems	Self-biting hyperactive	Aggressive anxiety	−	−	+	Hyperactive attention-deficit	Aggressive, attention-deficit, hyperactive	Hyperactive	−	−
Seizures	−	+	+	+	−	−	+	+	−	−
Syndrome diagnosis							Lennox–Gastaut			
Neurological exam	Hypotonia	Spastic quadriplegia	Axial hypotonia, lower limb spasticity	Hypotonia, lower limb spasticity	Normal	Normal	Normal	Normal	Hypotonia	Hypotonia
Growth retardation, prenatal	−	−	−	−	−	−	−	−	−	−
Growth retardation, postnatal	+	+	+	+	−	−	−	−	+	+
Obesity	−	−	−	−	−	−	+	+	−	−
Microcephaly	+	−	+	+	−	−	−	−	−	+
Dysmorphisms	+	+	+	+	−	−	−	?	+	+
Joint laxity	−	−	−	−	−	−	−	?	−	−
Hypotonia	+	+	+	+	−	−	−	?	+	+
Additional medical problems	GER	GER, gastric ulcer	GER	GER gastrostomy	−	Bulimia	GER	Bulimia	GER	Nephrotic syndrome, central hypothyroidism
MRI brain	Small brain, mild enlargement of sulci in frontal lobes	Moderate brain atrophy	?	?	Normal	Normal	Normal	?	Normal	Normal

If features are not described in the original article, we assume they are not present. Note that more details on the female phenotypes were available in some cases.

−, absent; +/−, mild; +, moderate; ++, severe; ?, unknown. GER, gastroeosophageal reflux; ID, intellectual disability; IDDM, insulin-dependent diabetes mellitus; n.a., not applicable; PFO, patent foramen ovale; XCI, X-chromosome inactivation.

**Table 3:** Clinical description of female cases presented; detailed epilepsy phenotypes

Patient	1	2	3	4	5	6	7	8	9	10	11	12	13	14	Ref. 9	Ref. 10	Ref. 11
Age	26	9	25	11	36	2,5	9	18	5,5	2,3	53	11	51 (deceased)	8,5	26	13	17
Mutation	c.4185del p.Lys 1396fs	c.438 C>A p.Cys 146*	c.2042del p.Gly681fs	c.964 C>T p.Arg322*	c.2201_2202 delAA p.Lys734 Serfs*24	c.1441 C>T p.Arg481*	c.3053-3066 del14 p.Gly1018 Aspfs*2	del exon1	c.1582delA p.Arg 528Glufs*4	c.1882C>T p.Arg 628*	c.2725del p.Ala909 Profs*13	c.652 C>T p.Arg 218*	c.952C>T p.Gln 318*	c.3596_3597 insA p.Lys 1199Asnfs	46,X,t (X;3) (q13;q11)	c.1882 C>T p.Arg 628*	c.964C>T p.R322*
XCI	57:43 (random)	70:30 (random)	52:48 (random)	63:37 (random)		100% skewing		62:38 (random)	64:36 (random)						100% skewing	65:35 (random)	73:27 (borderline skewed)
*KIAA2022* expression (ratio)	0,000624456		0,00029598	0,000355424	0,000256076	Absent									absent		
Degree of ID	+/− to +	+	+/−	+/−	+ to ++	+	+/− to +	+ to ++	+	+/−	−	+	++	+	++	+/−	++
Seizures	+	+	+	+	+	−	+	+	+	−	+	+	+	+	−	−	+
Age seizure onset (months)	8	8	72	30	36		24	24	14		192–216	24	84	36			18
Generalised	+	+	+	+	+	−	+	−	+	−	+	+	+	+	−	−	+
Myoclonic	+	+	+	+	+	−	+	−	+	−	?	+	+	+	−	−	?
Typical absence	−	+	+	−	+	−	+	−	+	−	+	+	−	−	−	−	+
Myoclonic absence	−	−	+	−	−	−	+	−	+	−	?	−	−	+	−	−	?
Tonic	−	−	−	+	−	−	−	−	+	−	−	−	−	+	−	−	+
Atonic	−	+	−	+	−	−	−	−	+	−	−	−	+	+	−	−	+
Clonic	−	−	−	−	−	−	−	+	−	−	−	+	−	+	−	−	−
GTCS	+	+	+	−	+	−	−	−	+	−	−	+	+	−	−	−	+
Focal	−	+	−	+	Probably not	−	−	+	−	−	−	+	−	−	−	−	−
Spasms	−	−	−	−	−	−	−	−	−	−	−	−	−	−	−	−	−
Status epilepticus	−	+	−	?	+	−	?	+	?	−	?	+	+	−	−	−	?
Other seizure types	−	−	−	−	−	−	Automatisms (focal seizures?)	−	−	−	?	−	−	−	−	−	?
Photosensitivity	−	+	+	?	−	−	−	−	?	−	−	−	?	−	−	−	?
EEG	PSW	PSW, ECS	PSW, ECS	PSW, focal discharges	Generalised SWC and PSW	Normal	PSW	PSW, right focal discharges	Background slowing and generalised and multifocal epileptiform discharges	Normal	?	Mixed generalised (PSW and slow wave) and focal discharges	Interictal: featureless; ictal: bifrontal PSW, fast activity, evolving into diffuse slow rhythmic activity; ECS	Interictal: generalised SWC and PSW; ictal: PSW concomitant with palpebral clonia or atonic fall	?	?	Suggestive of multifocal and generalised epilepsy
Seizure outcome	Ongoing despite AED	Ongoing despite AED	Ongoing despite AED	Ongoing despite AED	Ongoing despite AED	n.a.	Ongoing despite AED	Ongoing despite AED	Ongoing despite AED	n.a.	Ongoing despite AED	Ongoing despite AED	Ongoing until death despite AED	Ongoing despite AED	n.a.	n.a.	Ongoing despite AED

−, absent; +/−, mild; +, moderate; ++, severe; ?, unknown. AED, anti-epileptic drugs; ECS, eye-closing sensitivity; GTCS, generalized tonic-clonic seizure; ID, intellectual disability; n.a., not applicable; PSW, polyspike waves; SWC, spike wave complex; XCI, X-chromosome inactivation.

### Molecular analysis

#### *KIAA2022* mutations

**Figure 1:**
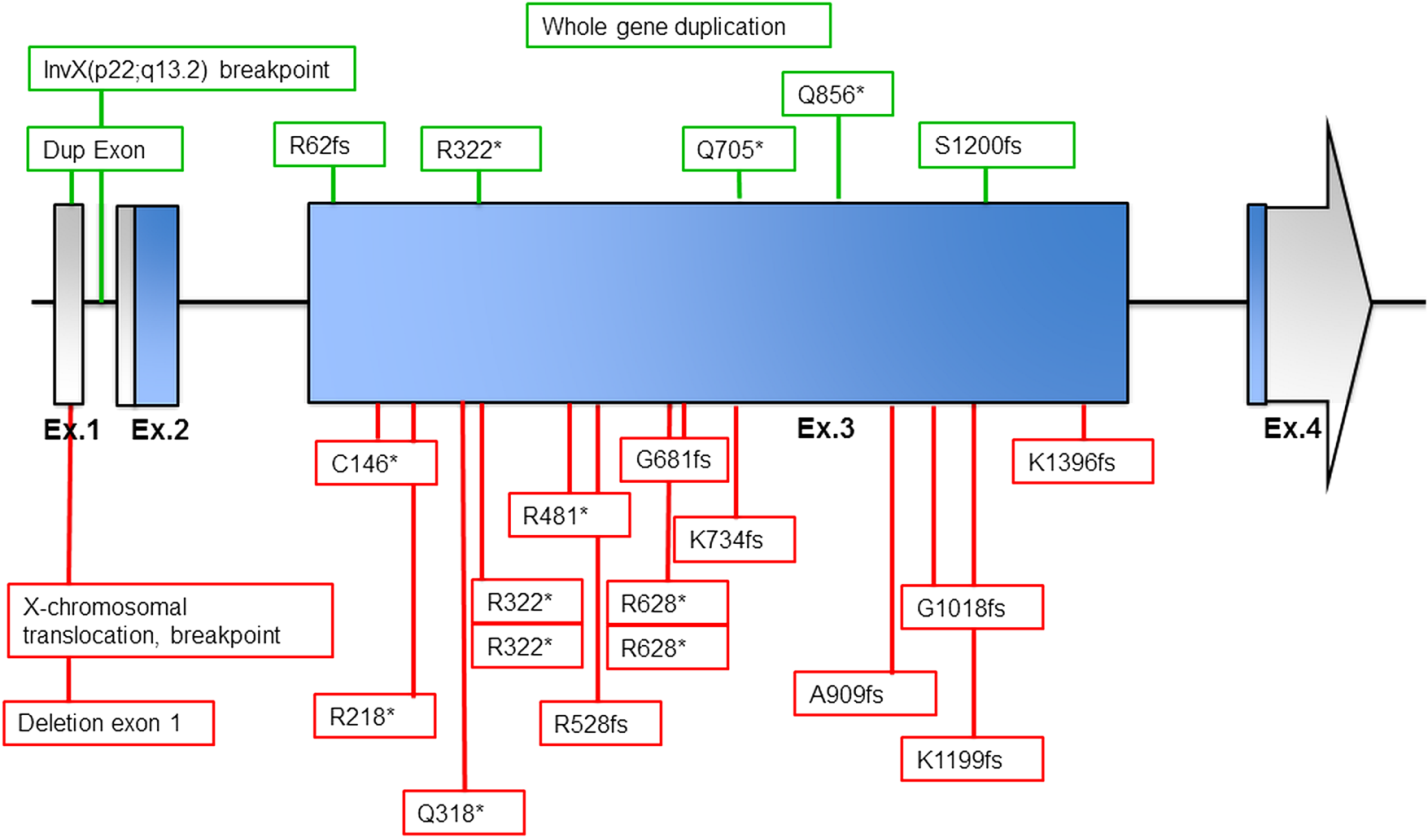
Genomic organisation of *KIAA2022* and location of mutations. Figure shows schematic presentation of known exon–intron organisation of *KIAA2022*. Exon size is at a scale apart from exon 4, for which the arrow indicates the continued size. Untranslated regions are indicated by grey colour, and the coding regions are indicated by blue colour. The boxes indicate the location and identity of the (previously) observed mutations in female (lower/red boxes) and previously reported mutations in male patients (upper/green boxes).

Thirteen patients carried a de novo truncating mutation in *KIAA2022* (NM_001008537.2) (table 1). In one patient, a deletion of the complete non-coding exon 1 was identified by array CGH, which is likely to affect the expression of the protein. Figure 1 represents the genomic organisation of the *KIAA2022* gene, as well as the location of the mutations reported previously and those presented here. All mutations were absent from the Exome Aggregation Consortium (ExAC) database (URL: http://exac.broadinstitute.org) (accessed October 2015).^17^ All sequence alterations are in the large exon 3 (coding exon 2) and are predicted to activate non-sense mediated decay (NMD), with one recurrent mutation, p.Arg322*, that is present in one of the currently described females (patient 4), one male patient^3^ and one previously reported female.^11^ Overall, there seems to be no particular domain in which mutations cluster, consistent with the hypothesis that these truncating.

mutations all cause LOF of *KIAA2022*, regardless of their position in the protein.

#### Chromosome X-inactivation studies and *KIAA2022* expression

**Figure 2:**
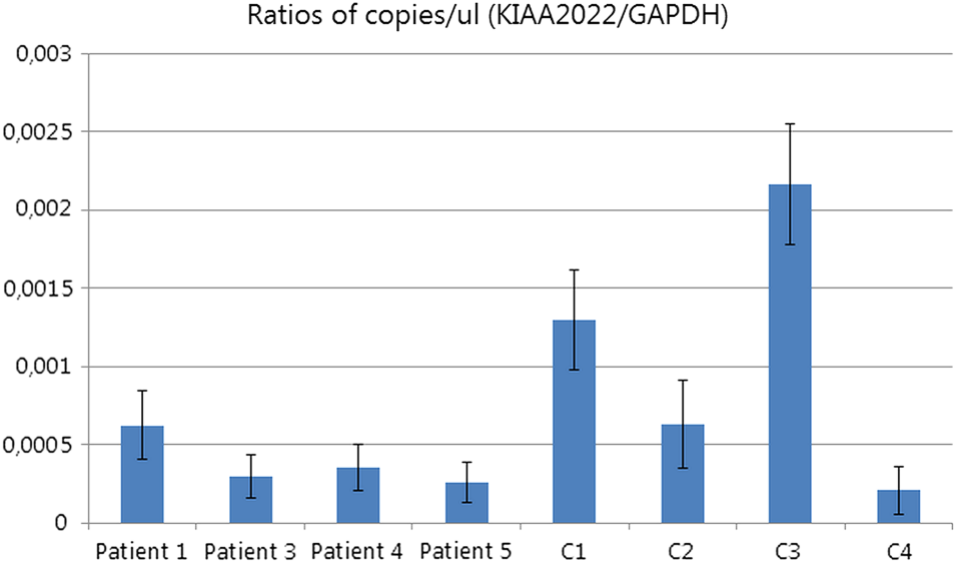
Relative expression of *KIAA2022* in four cases versus female controls. Y-axis gives the ratio of positive droplets for *KIAA2022* vs GAPDH; experiment was done in triplicate. 95% CIs are indicated by error bars.

X-inactivation was tested in seven patients and was found to be random in patients 1, 2, 3, 4, 8 and 9. One patient (patient 6) showed 100% skewing. We further tested *KIAA2022* expression using digital droplet PCR of RNA derived from whole blood of four patients (patients 1, 3, 4 and 5) and compared it with four healthy female controls. *KIAA2022* expression in blood was expressed as a ratio of *KIAA2022* mRNA copies compared with *GAPDH* and was found to be low in both cases and controls. Cases had on average a two to three times lower expression than female controls, but this was not statistically significant (non-parametric test on ratio of cases vs controls, p=0.486; figure 2). Expression of *KIAA2022* in patient 6 was tested using qPCR and was found to be completely absent.

### Previously described patients

A literature search resulted in 13 publications on *KIAA2022*. Of these, seven reported patients with sequence alterations of *KIAA2022* or structural X-chromosome abnormalities affecting *KIAA2022,*^1–3^ ^5^ ^9–11^ whereas the other six did not describe patients. In these first seven publications, 15 male patients were described. Clinical characteristics are given in table 2. Five affected males from one family^5^ are excluded from the table, because only limited clinical data were available. Three female patients affected by *KIAA2022* disruptions have previously been reported,^9–11^ two with a phenotype comparable with that in males, and one with mild ID. Clinical characteristics are given in tables 1 and 3.

### *KIAA2022* mutations in public databases

All mutations reported in the currently described females were absent from public databases. However, a few other truncation variants have been reported. In the ExAC database, three female and one male subjects out of a total of 60 706 subjects are reported to have heterozygous apparent LOF variants in *KIAA2022* (hg19, NM_001008537.2), namely, chrX:g.73959335T>C (c.4458-2A>G; one female, resulting in a putative disruption of the canonical splice acceptor site for the last exon), chrX:g.73959987G>A (c.4405C>T or p.Arg1469*; two females, located right at the end of the encoded protein) and chrX:g.73961129_73961139delCTCTCACATCT (p.Arg1085TyrfsTer45; one hemizygous male).

## Discussion

We report 14 female patients with a heterozygous de novo mutation of the X-chromosomal *KIAA2022* gene. Thirteen mutations resulted in a frameshift or premature stop codon that can elicit NMD, predicting a complete LOF of the protein. The 14th mutation is a complete deletion of the non-coding exon 1, most likely affecting the expression of the protein. *KIAA2022* mutations and alterations have previously been reported in males with severe ID,^1–3^ ^5^ and it is an established XLID gene. We show that *KIAA2022* mutations can also cause a phenotype in females. Overall, 11 of our female patients with *KIAA2022* loss-of-function mutations showed a similar clinical phenotype, which added to our belief that these mutations were indeed responsible for their phenotype. This phenotype was characterised by intractable epilepsy with predominant myoclonic seizures and/or absences, with onset in infancy or early childhood, behavioural problems and mild to severe ID. Developmental delay preceded epilepsy onset in six individuals. Hypotonia, neonatal feeding difficulties, microcephaly and mild dysmorphic facial features were less frequent findings.

Three female patients affected by *KIAA2022* disruptions were previously reported. Moyses-Oliveira *et al* reported a female patient with a balanced X-autosomal translocation disrupting *KIAA2022* at intron 1.^9^ XCI studies showed inactivation of the normal X-chromosome in all cells, leading to complete absence of *KIAA2022* expression. The reported phenotype was comparable to the previously described male phenotype, with severe ID, microcephaly, autistic behaviour, growth retardation and facial dysmorphia, without epilepsy. Athanasakis *et al*^10^ reported a 13-year-old girl with mild ID and a de novo non-sense mutation of *KIAA2022* (p.Arg628*) as part of a larger study which included patients with ID and absence of dysmorphic features, normal growth parameters and no seizures or malformations. The X-inactivation pattern was 65:35. Farach and Northrup^11^ reported a 17-year-old girl with a recurrent de novo non-sense mutation of *KIAA2022* previously reported in a male (p.Arg322*) and a phenotype comparable to previously reported male patients, with severe ID, hypotonia, behavioural problems, microcephaly, growth retardation, mild dysmorphisms and seizures. An X-inactivation pattern of 73:27 was reported. These previously reported female patients seem to be on the extreme ends of the phenotypic spectrum in comparison to our new cases, with one patient being relatively mildly affected,^10^ while the other two have a phenotype comparable to our most severely affected cases.^9^ ^11^ Although we observe a comparable clinical picture in most female cases, this illustrates that the phenotypes of females affected by *KIAA*2022 mutations can be very variable. Moreover, unaffected female carriers of *KIAA2022* disruptions have been reported,^2^ and the ExAC database includes three females with heterozygous apparent LOF variants in *KIAA2022.*^17^

No inheritance or clinical data are available for the females in the ExAC database, but it does not include patients with paediatric onset ID.^17^ However, the presence of these variants in the ExAC database might be explained by the nature of the variants. Regarding the truncating alteration (Arg1469*), it appears that this alteration will not undergo non-sense mediated decay, as it is located at the end of the second to last exon. Therefore, a truncated protein, lacking the last 48 amino acids, will be produced, which may not be a complete LOF alteration. The splice alteration is in the splice acceptor in the last exon, and it might have minimal functional impact since it is localised near the 3′-end of the protein. The apparent hemizygous frameshift variant (Arg1085fs) looks like an in-frame indel that was miscalled (c.3245_3265del21insCCT; deletion of 21 bp, insertion of 3 bp) upon manual visualisation. Therefore, these reported variants are likely non-pathogenic. Furthermore, it is worth noticing that ExAC variants are not validated, and some of them could be false positives. On the other hand, it is possible that these mutations are pathogenic but that they occur in asymptomatic female carriers, as previously described.^2^

Although both males and females with *KIAA2022* mutations have ID and frequent autistic behaviour, males tend to have a more severe phenotype. They have more severe ID (as opposed to mild to severe in females) and more frequently microcephaly, hypotonia, growth retardation, feeding difficulties and facial dysmorphisms. Epilepsy was reported in 5 of 10 male patients, whereas it was seen in 13 of 17 females, making it a more frequent clinical finding in females.^1–3^ It should be noted, however, that some of our female patients (1, 2, 3 and 5) were selected for genetic testing because of their epilepsy, which may have introduced a selection bias.

A reduced penetrance and variable expression in females and a more severe phenotype in males versus females is a common observation in many X-linked disorders, and may be explained by XCI patterns in females.^18–22^ Our female patient 6 and the patient previously reported by Moyses-Oliveira *et al*^9^ support this theory. Both had 100% skewed X-inactivation patterns and complete loss of expression, leading to a phenotype closely resembling the more severe male phenotype. Conversely, a previously reported male with the least severe phenotype among reported males was shown to have a duplication of exon 1, resulting in a decrease in expression of 60%.^2^ This is in contrast to several other male patients that lack expression completely. So far, the degree of *KIAA2022* loss thus seems to correlate with the severity of the phenotype, with complete loss of expression predicting a severe phenotype. The patient reported by Farach and Northrup^11^ had a borderline skewed X-inactivation (73:27) and was also severely affected. In six out of seven tested female patients, a random XCI was found, and expression of *KIAA2022* was on average two to three times lower than that in female controls, although this was not statistically significant. These female patients all showed a similar clinical phenotype, characterised by intractable myoclonic epilepsy and an ID that is less severe than in male patients. The very mildly affected patient described by Athanasakis *et al*^10^ also had a random X-inactivation according to our criteria (65:35). These results indicate that partial loss of *KIAA2022* expression explains the phenotype in those females who are more mildly affected than males. Unfortunately, data on X-inactivation patterns are lacking in patient 11, who has a very mild phenotype compared with other affected females, and in the unaffected female carriers of familial *KIAA20022* disruptions. However, expression in at least one unaffected female carrier was reported to be normal compared with controls, suggesting XCI skewing towards the wild-type allele as an explanation for their lack of symptoms.^5^ No clinical data on unaffected female carriers are available.

Next to X-inactivation patterns in blood, other factors might also explain the clinical variability in females with *KIAA2022* mutations. First, X-inactivation and expression in blood might not reflect what is occurring in the brain. Second, other factors may modify expression, such as variants in regulatory sequences of *KIAA2022* and related genes, or a parent of origin effect. Finally, mechanisms other than loss of expression might also play a role in affected females. The random XCI found in several female patients predicts a mosaic population of cells with either normal or absent expression of *KIAA2022*, in contrast to male patients, where all cells have the defective allele. Although this mosaic cell population in females may have similar effects as the defect in males, an additional disease mechanism might be cellular interference, as is the proposed disease mechanism in *PCDH19*-related epilepsy.^23–26^ In females, *PCDH19* mutations cause variable degrees of epilepsy, behavioural problems and intellectual deficits, while male carriers are unaffected. However, one reported male with a female phenotype was found to be mosaic for a *PCDH19* mutation, just as affected females.^25^ Brain mosaicism in females, as a result of random X-inactivation, was proposed to disrupt cell–cell interactions between the two different cell populations (the cells with normal *PCDH19* on the one hand, and the cells with mutated *PCDH19* on the other). A similar disease mechanism is plausible, since the *KIAA2022* protein seems to be involved in the same processes of cell–cell and cell–matrix adhesion and neuronal migration as the *PCHD19* protein, by influencing expression of N-cadherin.^7^ Additional X-inactivation and expression studies of asymptomatic mothers of affected males carrying *KIAA2022* mutations could further clarify the disease mechanism in females with *KIAA2022-*related disease.

## Conclusion

In conclusion, we show that de novo truncating mutations in the X-chromosomal *KIAA2022* gene can lead to a phenotype in males and in females. While males had more pronounced ID and dysmorphic features, females with *KIAA2022* mutations show variable symptoms, and some are even asymptomatic. Females with 100% XCI skewing and absent *KIAA2022* expression show a phenotype similar to the male phenotype. Females with random XCI patterns tend to have a more prominent epilepsy phenotype, with predominant generalised myoclonic and/or absence seizures. Mechanisms underlying the female phenotype may be both cellular mosaicism and reduced protein expression.

## Footnotes

i Patients 7 and 12 are also being described in a manuscript currently under review: De novo loss of function mutations in *KIAA2022* are associated with seizures and developmental delay in females by Rachel Webster, Megan T. Cho, Kyle Retterer, Francisca Millan, Catherine Nowak, Jessica Douglas, Ayesha Ahmad, Amber Begtrup, Dianalee McKnight, Orrin Devinsky, Wendy K. Chung.
