# Supplementary material for: De novo mutations of KIAA2022 in females cause intellectual disability and intractable epilepsy
Source: J Med Genet. 2016 Jun 29;53(12):850–8. doi: 10.1136/jmedgenet-2016-103909 (PMC5264224; doi:10.1136/jmedgenet-2016-103909)
Supplement: Supplementary material [file jmedgenet-2016-103909supp.pdf]

Supplemental material

Appendix 1: molecular analysis

Whole exome sequencing:

Three patients (patient 1, 2 and 3) and their parents were sequenced using SureSelect XT Human All Exon V5 kit (Agilent) enrichment and the HiSeq2500 sequencing system (Illumina) at a mean target depth of 100X. Reads were aligned to Hg19 using BWA (BWA-MEM v0.7.5a) and variants were called using the GATK haplotype caller (v2.7-2). Detected variants were annotated, filtered and prioritized using the Bench lab NGS v.3.1.2 platform (Cartagenia, Leuven, Belgium). Sequencing and analysis was performed at the diagnostics section of the Department of Medical Genetics, University Medical Center Utrecht, The Netherlands.

Whole exome sequencing and data analysis of patient 4 and her parents was performed as described previously [1], at the Wellcome Trust Sanger Institute (Hinxton, Cambridgeshire). In brief, genomic DNA (~3 mg) was fragmented by sonication, and fragments with a length of 150–200 bp were purified. After a paired-end DNA library was prepared from the DNA fragments (with the TruSeq DNA Sample Preparation Kit from Illumina), targeted enrichment was performed with the SureSelect Human All Exon 50Mb Kit (Agilent Technologies). Captured DNA was then sequenced on a HiSeq2000 (Illumina) as paired-end 75 bp reads according to the manufacturer's protocol.

Whole exome sequencing of patient 6 and her parents was conducted through Ambry Genetics using paired-end, 100 cycle chemistry on the Illumina HiSeq 25000. Enrichment was performed using SeqCap EZ VCRome 2.0 (Roche NimbleGen, Madison, WI). Data were annotated with the Ambry Variant Analyzer tool, including nucleotide and amino acid conservation, biochemical nature of the amino acid substitutions, population frequency, and predicted functional impact as

previously described .[2] Approximately 94% of the bases sequenced had a base calling accuracy of 99.9% and 95% of patient's exome was covered at 10x or higher.

Clinical whole exome sequencing of patient 7 and 12 was performed by GeneDx. Genomic DNA was extracted from whole blood from the affected children and their parents. Exome sequencing was performed on exon targets isolated by capture using the Agilent SureSelect Human All Exon V4 (50 Mb) kit (Agilent Technologies, Santa Clara, CA). The sequencing methodology and variant interpretation protocol used has been previously described.[3] The general assertion criteria for variant classification are publicly available on the GeneDx ClinVar submission page (http://www.ncbi.nlm.nih.gov/clinvar/submitters/26957/).

Diagnostic whole exome sequencing of patient 10 and her parents was performed at Mendelics Genomic, Sao Paulo Brazil) on a DNA sample extracted from peripheral blood. The Extended Nextera Rapid-Capture Exome kit was used and sequencing was performed on the Illumina HiSeq2500 system (Illumina, San Diego, CA, USA). Exome reads were analyzed in a standard Bioinformatics pipeline, using BWA for sequence alignment to the GRCh37 reference, Broad Institute GATK for genotyping, SnpEff for variant annotation, and ExomeDepth for CNV detection.

Patient 13 and her parents were exome sequenced at the Epilepsy Society, Chalfont, using the Ion AmpliSeq Exome RDY Kit (ThermoFisher) and the Ion Proton sequencing system (LifeTechnologies). Mean target depth of >100X was obtained for each member of the trio (Mother 139X, Father 163X, Proband 101X). Variants were called and Trio analysis was undertaken using Ion Reporter software Ampliseq Exome Trio v5.0 workflow.

For patient 14 and her parents, library generation, exome enrichment and clinical WES were performed at the French National Centre for Genotyping (CNG, Evry, France). Briefly, libraries

were prepared from 3 µg genomic DNA extracted from whole blood using an optimized SureSelect Human Exome kit (Agilent) following the manufacturer's instructions. Captured, purified and clonally amplified libraries targeting the exome were then sequenced on a HiSeq 2000 (Illumina) according to the manufacturer's recommendations. Obtained sequence reads were aligned to the human genome (hg19) using BWA software. Downstream processing was carried out with the Genome analysis toolkit (GATK) [4,5], SAMtools [6] and Picard Tools (http://picard.sourceforge.net/). Single-nucleotide variants and indels were subsequently called by the SAMtools suite (mpileup, bcftools, vcfutil). All calls with a read coverage ≤5x and a Phred-scaled SNP quality of ≤20 were filtered out. Substitution and variation calls were made with the SAMtools pipeline (mpileup). Variants were annotated with an in-house Paris Descartes bioinformatics platform pipeline based on the Ensembl database (release 67) .[7]

Whole genome sequencing of patient 9 was done in a research setting at Complete Genomics (Mountain View, CA) as described previously.[8] Variants were annotated using Annovar and custom script at The Centre for Applied Genomics (TCAG, Toronto, Canada).

Sequencing for patient 5 was performed in a research setting at the University of Washington (Seattle, WA, USA). All library preparation, data analysis, and variant calling were performed as described previously .[9] Briefly, all coding *KIAA2022* exons and at least five base pairs of flanking intronic sequences were captured by using molecular inversion probes (MIPs); next-generation sequencing was performed used a 100 paired-end protocol on the Illumina HiSeq, reads were aligned to Hg19 using BWA (v0.7.8) and variants were called with GATK UnifiedGenotyper (v2.4-9).

All mutations were confirmed by standard Sanger sequencing.

Patient 8 was diagnosed by Array-CGH using the Agilent 180 k chip (Arr[hg19]Xq13.3(74,108,610-74,178,971)x1

Patient 11 was diagnosed by targeted analysis of the *KIAA2022* mutation identified in her two sons.

X-inactivation studies:

X-inactivation ratios were determined in patient 1, 2, 3, 4, 6 and 9 by methylation-sensitive restriction digest (CfoI) of genomic DNA followed by PCR and fragment analysis of the highly polymorphic trinucleotide repeat within the first exon of the human androgen receptor gene (AR), to distinguish between the maternal and paternal alleles and simultaneously determine their methylation status. A methylation ratio of up to 1:4 is considered normal.

Expression studies:

Expression of KIAA2022 was evaluated in patient 1, 3, 4 and 5, with digital PCR using Taqman probe Hs02339405_m1 (Invitrogen) spanning exon 2-3. GAPDH dHsaCPE5031597 (Bio-Rad) was used as an endogenous control. Droplets were generated in a QX100TM and thermal cycling was done using a Bio-rad C1000 Touch Thermal Cycler. A QX200 Droplet Reader and QuantasoftTM Software were used for data acquisition and analysis. Expression levels of cases and female controls were compared using the Poisson corrected ratios of copies/ul (KIAA2022/GAPDH) as implemented in the QuantasoftTM Software.

References

1. Suls A, Jaehn J, Kecske A, et al. De Novo Loss-of-Function Mutations in CHD2 Cause a Fever-Sensitive Myoclonic Epileptic Encephalopathy Sharing Features with Dravet Syndrome. *Am J Hum Genet* 2013;**93**:967–975.
2. Farwell KD, Shahmirzadi L, El-khechen D, et al. Enhanced utility of family-centered diagnostic exome sequencing with inheritance model – based analysis : results from 500 unselected families with undiagnosed genetic conditions. *Genet Med* 2015;**17**:578–586.
3. Tanaka AJ, Cho MT, Millan F, Juusola J, Retterer K, Joshi C, Niyazov D, Garnica A, Gratz E, Deardorff M, Wilkins A, Ortiz-Gonzalez X, Mathews K, Panzer K, Brilstra E, van Gassen KL, Volker-Touw CM, van Binsbergen E, Sobreira N, Hamosh A, McKnight D, Monaghan KG, Chung WK. Mutations in SPATA5 Are Associated with Microcephaly, Intellectual Disability, Seizures, and Hearing Loss. *Am J Hum Genet* 2015;**97**:457-64.
4. McKenna A. et al. The Genome Analysis Toolkit: a MapReduce framework for analyzing next-generation DNA sequencing data. *Genome Res* 2010;20:1297–1303.
5. DePristo MA et al. A framework for variation discovery and genotyping using next-generation DNA sequencing data. *Nat. Genet* 2011;43:491–498.
6. Li H et al. The Sequence Alignment/Map format and SAMtools. *Bioinformatics* 2009;25: 2078–2079.
7. Flicek P et al. Ensembl 2012. *Nucleic Acids Res* 2012;40:D84–D90
8. Drmanac R, Sparks AB, Callow MJ, et al. Human Genome Sequencing Using Unchained Base Reads on Self-Assembling DNA Nanoarrays. *Science* 2010;**327**:78-81.
9. Carvill GL, McMahon JM, Schneider A, et al. Mutations in the GABA Transporter

SLC6A1 Cause Epilepsy with Myoclonic-Atonic Seizures. *Am J Hum Genet* 2015;**96**:808–815.

Appendix 2: clinical descriptions

Patient 1 (Dutch)

This 26 years old female was diagnosed with epilepsy at the age of 8 months, but in retrospect she may have had seizures already in the first months of life. She has therapy resistant myoclonic seizures, that used to occur up to 30 times per day, but seizure frequency has gradually decreased to 4 per day. Except for two tonic-clonic seizures, she has had no other seizure types. EEGs showed generalized (poly)spike-wave complexes. Brain MRI at age 20 showed no abnormalities. Psychomotor retardation became evident at age 18 months. She could walk independently at age two years and speech development started at age three years. She currently has a mild to moderate intellectual disability (IQ ~50) with an estimated developmental age of 3 years. An autism spectrum disorder has been diagnosed. Furthermore, her medical history includes congenital hip dysplasia, joint laxity and obesity. No facial dysmorphisms were observed.

Patient 2 (Dutch)

Seizure onset in this 9 years old female patient was at age 8 months. She presented with myoclonic seizures, but later also developed absences, myoclonic atonic seizures, atonic seizures and focal dyscognitive seizures, refractory to pharmacological treatment. She also had a single tonic-clonic seizure. In the first weeks of life she had 2 episodes with loss of muscle tone, apnea and cyanosis, which in retrospect might have been seizures too. At the age of 2 years and 9 months she developed a myoclonic status epilepticus. She self-induces seizures by looking at lights. Her EEG at age 5 showed slight background slowing with sporadic generalized spike wave complexes when awake. During sleep frequent spike wave complexes in the temporal regions, and frequent generalized polyspikes were seen. Her last EEG at age 8 also showed eye-

closure related discharges. Brain MRI at age 5 showed no abnormalities. Her psychomotor development slowed after the onset of her seizures. At age 5 years and 8 months she had a developmental age of 28 months. She shows hyperactivity, aggression and autistic behavior. In addition, her medical history includes severe esophageal reflux during her infancy, and obesity. She has a narrow forehead and a hypotonic face with an open mouth.

Patient 3 (Dutch)

This 25-years old female patient was diagnosed with epilepsy at age 6 years. In retrospect, her parents reported that she might have had myoclonic seizures already in her infancy. She developed myoclonic absences, absences and tonic clonic seizures, refractory to pharmacological treatment. Her EEG showed generalized polyspikes, epileptic discharges over the frontal regions and eye closure sensitivity. Brain MRI at age 19 was normal. Motor and speech development were delayed. She could walk independently at age 19 months. She had an IQ score of around 55. Her behavior is problematic with hyperactivity and tantrums. Additional medical problems consisted of neonatal feeding difficulties with persistent vomiting and obesity. No facial dysmorphisms were observed.

Patient 4 (Danish)

This patient is an 11 years old female with myoclonic atonic epilepsy, with onset of atonic seizures at age 2.5 years. She was born at 38 weeks with a birth weight of 2065 gram (<-2 SD). She had multiple seizure types, including myoclonic seizures, atonic seizures, focal seizures and tonic seizures, refractory to multiple antiepileptic drugs. She currently has daily atonic and tonic seizures. Her EEG showed generalized spike-wave complexes mixed with focal discharges. Her

development was normal prior to seizure onset, except for a slight language delay. She now has a moderate developmental delay. Repetitive behavior and ADHD have been reported.

Patient 5 (Belgian)

This patient is a 36 years old female who presented with myoclonic seizures at age 3 years. She soon developed frequent therapy resistant seizures consisting of myoclonic seizures, tonic clonic seizures, absences and non-convulsive or myoclonic status epilepticus. Seizure frequency often increased during the premenstrual period. EEG showed 2 Hz generalized spike waves, spikes and polyspikes. MRI of the brain was normal, as was ophthalmologic examination, skin biopsy, and evoked potentials. Early motor development was normal and she walked independently at the age of 14.5 months. Her first words were spoken at the age of 3 years. Since then, developmental delay became clearer and she now has a moderate to severe intellectual disability with hyperactive behavior.

Patient 6 (US)

This patient is a 2 years and 8 month old girl of Mexican descent, without any symptoms of epilepsy. Routine EEG was normal. Concerns regarding her development were first noted at 3 months of age, and at 2 years and 8 months of age she was non-verbal and not yet walking. She currently has a moderate developmental delay. She was born full term with a birth weight of 2600 gram. She experienced poor feeding with gastroesophageal reflux in the neonatal period and has since had poor growth and failure to thrive. Her height is below the 2nd percentile and weight is at the 6th percentile for her age. She has dysmorphic features characterized by microcephaly, hypotonic facies, narrow forehead, anteverted nares, open mouth, large ears,

upslanting palpebral fissures, hypertelorism, and overriding second toes bilaterally. Furthermore, she has severe generalized hypotonia. No behavioral problems are reported, especially no autistic features.

Patient 7(US)

Patient 7 is a 9 year old girl of unrelated Caucasian parents. She showed a normal developmental profile until the age of 2 years when her epilepsy started and her development started slowing. Her seizures were initially absence seizures and myoclonic seizures that have been difficult to control on multiple antiepileptic medications. At the age of 9 years, she mainly has absence seizures with associated head nods, but no myoclonic seizures or atonic seizures. She has an intellectual disability and pervasive developmental disorder with significant behavioral issues, currently at the developmental level of a 5 year old in all developmental domains. She never regressed or lost skills. Her general pediatric exam and neurological exam at the age of 9 years is unremarkable. She does not show dysmorphic features.

Patient 8 (French)

Patient 8 is a 17 year-old girl born to unrelated parents originating from Portugal, Italy and France. Delivery occurred at term and parameters were normal. Development was normal until the age of 15 months when she entered hospital for a febrile illness. Subsequently her developmental milestones started slowing. It is during a psychomotor delay work-up at two years that an abnormal EEG was noticed. She was placed on Valproate that was discontinued when aged 8 years. She walked without assistance at 24 months but did not develop language. Her stereotypic movements and behavioral disorders during infancy suggested an autism spectrum

disorder (ASD). Seizures recurred at 11 years with poor response to antiepileptic drugs. She had an episode of status epilepticus at the age of 16 years. She currently has a moderate to severe intellectual disability. Toilet training is not obtained and she expresses herself with grunting. Behavioral problems are reported with fits of hyperactivity. At clinical examination, there is an open mouth, overweight and no dysmorphic features. Her length and OFC are normal.

Patient 9 (Canadian)

This patient is a 6 year-old girl who developed normally until 14 months of age, when atypical absences were noticed as well as tonic seizures that led her to fall while attempting to walk. Subsequently, she had mainly myoclonic jerks and atypical absences until around age 3 when the predominant seizures became atonic seizures of the head and the whole body with frequent drop attacks. At the peak of her epilepsy she was having a jerk, stare or drop every 3 minutes. Presently, she has 2-10 of the above every 5 hours. She takes multiple AED's and follows a ketogenic diet, which substantially improved the seizures. She also takes cannabis oil, which was also associated with improvement. EEG shows background slowing and generalized and multifocal epileptiform discharges. She has continued to develop though very slowly, without regression. She walks and is potty-trained. She speaks approximately 150 words and is able to make 5-word phrases. Her thinking and speech are regularly interrupted by absence spells. She is hyperactive and has impulse-control difficulties.

Patient 10 (Brazilian)

This 2 year-old female, born to non-consanguineous Brazilian parents, presented with hypotonia and joint laxity in the context of global developmental delay. There are no signs of epilepsy. She

walked without assistance at 19 months and her first words were pronounced at 18 months. Currently she speaks around 5 words without elaborating phrases. Brain MRI and EEG were unremarkable

Patient 11 (French)

This 34 year-old female came to medical attention after a diagnosis of *KIAA2022*-related epileptic encephalopathy was made in her two sons with ID, through sequencing of an intellectual disability related-gene panel. She has had absences-type epilepsy starting at age 17 years, that responded, although incompletely, to levetiracetam and lamotrigine. Currently, she describes persistent occasional absence episodes, about once per month. She has no intellectual disability; she has a university degree. She also has insulin-dependent diabetes mellitus and horseshoe kidneys.

Patient 12 (US)

Patient 12 is a 12 years old child of unrelated parents. She was noted to have developmental delays (gross and fine motor skills, decreased eye contact, stereotypies) before age 20 months. Her first seizures were clusters of myoclonic seizures at age 2 years, 3 days after the MMR vaccine. She subsequently developed myoclonic and absence as well as focal and generalized clonic seizures. She has intellectual disability and developmental delay. She has had developmental regression in the setting of a marked increase in seizure activity. EEG shows mixed generalized (polyspike-and-slow wave) as well as focal discharges. Her seizures have remained refractory to more than ten antiepipleptic drugs, the ketogenic diet, and vagus nerve stimulation. Her currently behavioral function is at a 4 year age level in all domains. Her general

physical examination is normal. Apart from her cognitive and behavioral deficits, her neurological exam is remarkable for impaired fine and gross motor and coordination skills. There are no focal neurological findings.

Patient 13 (UK)

This lady died at the age of 51 years. Following a normal pregnancy, she was born at term by breech delivery and was thought to have suffered a degree of hypoxic brain injury. Her development was delayed: she walked at the age of 2, her language development was markedly impaired and she was never able to speak in sentences. Her seizure onset was around the age of 7 years, with myoclonic seizures that responded well to treatment with ethosuximide and phenytoin for about 16 years. At the age of 23, her seizure control deteriorated with increased frequency of myoclonic seizures, often occurring in clusters with onset of atonic and generalised tonic-clonic seizures. Her seizures became soon drug-resistant and she experienced status epilepticus aged 38 years. She had severe intellectual disability and, from the age of 40, was confined to a wheelchair because of concerns about falls. She had facial dysmorphism, with small hands and feet. Her latest brain MRI scan, aged 37, was unremarkable. Her latest EEG video-telemetry recording, aged 50, showed frequent subtle seizures, of the order of seventy per day, mostly myoclonic (associated on EEG with an increase in frontal fast activity, sometimes followed by rhythmic slow activity) and atonic seizures (associated with bifrontal polyspike evolving into diffuse rhythmic slow activity). The most likely syndromic classification was myoclonic-astatic epilepsy.

Patient 14 (French)

The patient is an 8years old girl born from unrelated parents of French and Portuguese origins. All developmental milestones were acquired with moderate delay: she walked steadily around age 18 months, acquired fine motor skills after 3 years and presented with a marked language delay. The first words appeared at 3 years of age, and simple sentences are possible since age 7 years. She attends school in a specialized structure and has moderate intellectual deficiency. She has some autonomy in daily life. She does not have dysmorphic features, and her physical examination is normal, except for hyperlaxity.

Epilepsy started at the age of three years, by the association of atypical absences, with drooling sometimes followed by atonic fall of the head. Myoclonic and astatic falls were also described. The epilepsy was resistant to more than 10 antiepileptic drugs, to steroids and to a ketogenic diet. Currently, the child still suffers from weekly seizures and some rare tonic seizures during sleep. Electroencephalograms (EEG) showed slowing of occipital basal rhythm (6-7 Hz), and nearly continuous generalized spike-waves and poly-spike-wave complexes. This pattern is asymptomatic or may be concomitant with eye-lid clonia and/or atonic head falls. Lately, tonic seizures were recorded. Some physiologic sleep patterns appeared in the last EEG (at age 8).

MRI shows a mild frontal atrophy.
